# INSPECTOR: free software for magnetic resonance spectroscopy data inspection, processing, simulation and analysis

**DOI:** 10.1038/s41598-021-81193-9

**Published:** 2021-01-22

**Authors:** Martin Gajdošík, Karl Landheer, Kelley M. Swanberg, Christoph Juchem

**Affiliations:** 1 Department of Biomedical Engineering, Columbia University Fu Foundation School of Engineering and Applied Science, 3227 Broadway, New York, NY 10027 USA; 2 Department of Radiology, Columbia University College of Physicians and Surgeons, New York, NY USA

**Keywords:** Design, synthesis and processing, NMR spectroscopy, Biomedical engineering

## Abstract

In vivo magnetic resonance spectroscopy (MRS) is a powerful tool for biomedical research and clinical diagnostics, allowing for non-invasive measurement and analysis of small molecules from living tissues. However, currently available MRS processing and analytical software tools are limited in their potential for in-depth quality management, access to details of the processing stream, and user friendliness. Moreover, available MRS software focuses on selected aspects of MRS such as simulation, signal processing or analysis, necessitating the use of multiple packages and interfacing among them for biomedical applications. The freeware INSPECTOR comprises enhanced MRS data processing, simulation and analytical capabilities in a one-stop-shop solution for a wide range of biomedical research and diagnostic applications. Extensive data handling, quality management and visualization options are built in, enabling the assessment of every step of the processing chain with maximum transparency. The parameters of the processing can be flexibly chosen and tailored for the specific research problem, and extended confidence information is provided with the analysis. The INSPECTOR software stands out in its user-friendly workflow and potential for automation. In addition to convenience, the functionalities of INSPECTOR ensure rigorous and consistent data processing throughout multi-experiment and multi-center studies.

## Introduction

In vivo magnetic resonance spectroscopy (MRS) enables the noninvasive measurement of small molecules from localized regions in living tissue, thereby providing a powerful tool to assess tissue metabolism and function. The repertoire of measurable chemical compounds makes MRS a versatile tool for researching living systems (Supplementary Material, S1) and characterizing clinical conditions, especially for longitudinal studies requiring repeated experimentation. For instance, proton (^1^H) MRS is applied to many central nervous system disorders, ranging from multiple sclerosis to cancer, that involve significant metabolic alterations in the concentrations of small molecules^1^. Despite the strong potential of MRS for cutting-edge biomedical research and clinical applications, deviations from optimal data quality, processing strategy or quantification pipeline reduces the metabolic information that can be extracted. Resulting systematic errors have been suspected to be the key contributing factors for limited multi-site reproducibility^2^.

Concentrations of metabolites measured with in vivo MRS are typically in the millimolar range (< 12 mmol/L or mM)^3,4^. The concentration of tissue or free water signal (e.g. cerebrospinal fluid), by comparison, is in the molar range (40–55 M)^5^. Due to the low overall Boltzmann spin magnetization, MRS is a low-sensitivity method, and a single measurement of metabolites suffers from low signal-to-noise ratio (SNR)^6^. MRS benefits from a magnetic resonance (MR) scanner with a strong and homogeneous static magnetic field (B_0_)^7^, a relatively large volume of interest (VOI), and signal averaging, to name a few.

MRS software provided by MR scanner vendors is generally limited in scope and function, potentially resulting in inconsistent quantification accuracy and non-standardized study outcomes^8,9^. Data of high experimental quality should therefore be complemented by efficient and state-of-the-art data processing and analysis routines^9^. Robust and precise signal processing and analysis MRS are of the utmost importance because retaining every possible bit of information from start to finish is vital for accurate analysis of the final result. Similarly, potential erroneous and artificial signal contributions need to be identified and corrected or excluded from further analysis to avoid degradation or falsification of the derived metabolic information.

MRS experiments capture complex multi-dimensional data that are recorded as complex signal amplitudes over time in free induction decays (FID). An FID dataset can be defined as: N_R_ × N_Rec_ × N_FID_, where N_R_ is number of repetitions, N_Rec_ is number of receivers or coil channels, and N_FID_ is number of complex points in the FID. The FID signal recorded over the time domain (in msec) contains the sum of responses from all nuclei (spins) and needs to be transformed to the frequency domain (in Hz) with discrete Fast Fourier Transform (FFT) to constitute a spectrum. Although the frequency spectrum is the desirable form for interpretability, inspecting both time and frequency domains is beneficial for data processing.

Linear combination modeling (LCM) is the most popular method of quantifying the small-molecule metabolite concentrations expressed by in vivo MR spectra^9^. This method depends on high-quality basis functions considering the MRS experiment at hand and need to be measured or simulated for each metabolite. Novel MRS simulation algorithms are precise and fast^10^, and in combination with modern desktop workstations, simulation of the collection of basis functions (basis set) has become the preferred method over measurement in most laboratories. After analyzing all the information from the spectra, MRS signals from each compound in the sample can be quantified in arbitrary, institutional or absolute units (commonly in mM)^11^.

The typical MRS data processing stream often requires the scripted combination of several software packages, which are rarely computer platform-independent. An unmet need therefore exists for a comprehensive software tool offering integrated MRS signal processing, simulation and analysis.

The MRS software INSPECTOR was developed as a user-friendly and free package with all tools necessary for the many steps involved in translating raw FIDs from MRS experiments into physically interpretable metabolite concentrations. These include visualizing numerous aspects of the raw experimental data, processing the averaged spectrum, simulating the basis set used for linear combination modeling, and finally analyzing the processed spectrum with the basis set and LCM parameters of choice. The program is designed to enable the user to visualize, deeply inspect and manipulate each step of the workflow in a one-stop-shop interface. This paper highlights INSPECTOR’s key features most used in MRS research.

## Software architecture

### Platform-independent freeware tool

INSPECTOR was developed in MATLAB (MathWorks, Natick, MA) as a stand-alone user-friendly software with powerful analytic capabilities for applying magnetic resonance spectroscopy data to biomedical research and ultimately clinical applications^12^. INSPECTOR is freeware for non-commercial applications and requires neither MATLAB nor other software dependencies^13^. INSPECTOR is available for Microsoft Windows, Apple MacOS and Linux, making it platform-independent and functional on all commonly used operating systems. The only requirement is installation of the MATLAB Runtime environment available for download from the MathWorks website free of charge and without a license^14^.

### Interface and data management

**Figure 1:**
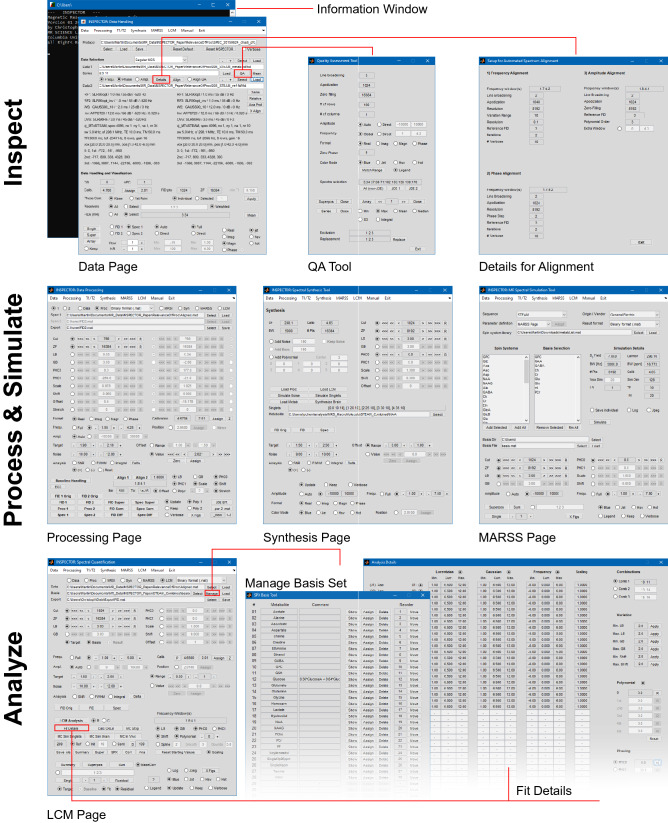
Graphical user interface in INSPECTOR for Microsoft Windows 10. The pages can be selected on the menu bar, and data can be transferred between pages.

INSPECTOR is organized into ‘pages’, each offering a set of functions related to specific aspects of MRS signal manipulation, like data handling, processing, simulation, and quantification. INSPECTOR is controlled entirely through a graphical user interface (GUI) (Fig. 1). The application pages are sorted in a menu bar from left to right according to a typical workflow. Additional information is either printed as a summary or in detail (“Verbose” mode) in an information window. Loaded data can be transferred from one page to another. The data can be accessed and visualized in either time or frequency domain on any page.

**Figure 2:**
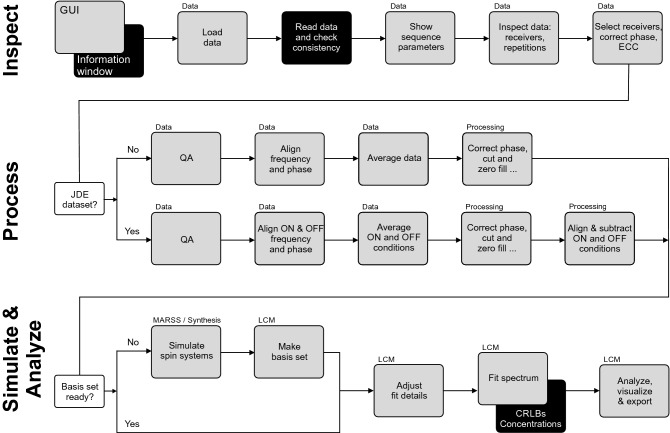
Typical processing pipeline in INSPECTOR for brain MRS. All steps are completely GUI based and no scripting or interfacing is required. Gray rectangles represent tabs on the GUI with page names in the upper left. Black rectangles represent the information window in steps when it is advised to pay attention to text output. *CRLB* Cramér–Rao lower bound, *ECC* eddy-current correction, *LCM* linear combination modeling, *JDE* *J*-difference editing, *MARSS* Magnetic Resonance Spectrum Simulator, *QA* quality assessment.

A typical processing workflow of brain MRS data in INSPECTOR comprises several steps, which can be further differentiated according to regular, *J*-difference editing (JDE) or other spectroscopy experiment types^15^ (Fig. 2). Experimentally acquired raw data are loaded and averaged on the Data page. Spectra can be further improved on the Processing page using standard signal processing methods. Basis sets for metabolite quantification can be simulated on the Synthesis or MARSS page and used for spectral analysis on the LCM page. An overview of each page follows below.

## Pages and applications

Each page, named for its corresponding step in the MRS data handling pipeline, contains various associated functions for data processing and visualization. Users can view function hints via mouseover. An overview of these functions is available in Supplementary Material (S2).

Examples from our and other laboratories are given below demonstrating particular functionalities available on each page of INSPECTOR. Examples from published studies are cited in the text; acquisition details can be found in Supplementary Material (S3).

### Data page

The Data page contains methods for loading and saving protocol files, loading data, quality assessment (QA), and spectral alignment for averaging individual traces into a single spectrum used in further processing and analysis.

Protocol files enable saving a complete image of all parameter selections and the INSPECTOR workflow. One file can store all adjustments and paths used for loading data, processing, simulation and quantification. Protocol files were designed to facilitate consistency and reproducibility of data processing, especially in case of data from longitudinal, multi-center, or otherwise large studies.

Data can be sorted according to MRS experiment type. A simple MRS experiment can be loaded as “Regular MRS”, whereas JDE experiments with two or three conditions can be loaded as “JDE”. INSPECTOR supports the following data formats:

- Siemens: .dat, .rda, .IMA, .dcm
- General Electric: .7
- Philips: .raw, .sdat
- Bruker: fid, rawdata.job0
- Varian: fid
- DICOM^16^: .dcm, .IMA

All MRS-relevant header information, such as echo time (T_E_), repetition time (T_R_), N_R_, spectral width, Larmor frequency, volume of interest (VOI) and more, are displayed on the Data page after the data are loaded. The Data page allows inspection of acquired receive channel-specific signals, which can be visualized as FIDs in the time domain or as spectra in the frequency domain. In vivo MRS experiments are susceptible to system B_0_ drifts, which have consequences for data quality as they may affect the efficiency of frequency-selective RF pulses for water suppression or editing, and cause spectral line broadening when MRS signals are combined. It is therefore desirable to break down long MRS scans into smaller parts to allow intermittent experimental frequency adjustments that mitigate such effects. INSPECTOR supports the handling and concatenation of such scan series in addition to basic single-scan data formats. Water references support the calculation of channel-specific scaling factors and phases for sensitivity-weighted channel combination of FID traces. Phase correction is based either on Klose’s method (used also for eddy-current correction, ECC)^17^, which is recommended, or on taking only the first FID point as a phase reference. Similar to the metabolite data, the header information is displayed as well.

In vivo MRS also commonly suffers from oscillatory frequency and phase variations due to magnetic susceptibility-induced B_0_ variations across the subjects’ respiratory cycles^18^. Before further treatment of signal repetitions, data can be inspected by the QA tool. The QA tool provides simple and fast data visualization and quality management for assessing frequency drift, hardware instability, subject motion, and other effects that can cause variability across scan repetitions. Visualization of the spectra in the QA tool enables the analysis and display of the signal (1) minimum, (2) maximum, (3) mean, (4) median, (5) standard deviation (SD) and (6) integral over the selected spectral frequency window. Erroneous data, e.g. due to technical malfunction of individual receivers or excessive motion, can be thereby identified and discarded. The QA tool can be applied before and after potential frequency, phase and amplitude corrections for optimal quality management and a side-by-side comparison of the effects of data manipulation and correction.

Frequency, phase or amplitude corrections are fully automated and based on an arbitrary number of concomitantly considered frequency windows (e.g. 0…2.1 ppm, 3.3…4.1 ppm and 7.1…9.1 ppm) and tailored spectral preprocessing such as line broadening, apodization (cut) and zero-filling. Frequency, phase and amplitude alignment rely on the comparison of a MRS trace with a reference, and a customized amplitude-weighted cross-correlation algorithm is applied to minimize the deviation of the two. The reference FID signal can be either a specific trace or a combination of traces (for improved SNR). Additional parameters include variation range and resolution in Hz for frequency alignment, phase step in degrees for phase alignment and polynomial order for amplitude alignment.

MR spectra pertaining to different JDE conditions, i.e. with editing radiofrequency (RF) pulses ON vs. OFF, are inherently distinct in content and thus also shape. INSPECTOR applies all corrections in an editing condition-specific fashion, before MRS traces are combined into two averaged spectra representing the ON and OFF conditions.

### Example: loading and pre-processing of brain MRS data

**Figure 3:**
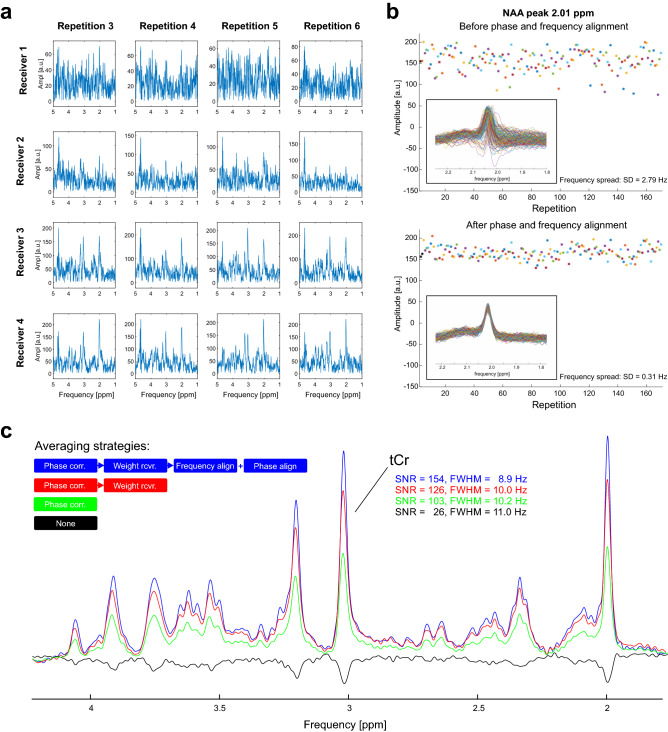
Data inspection and preprocessing in INSPECTOR. (**a**) Visualization of all individual repetitions from all coil channels in magnitude mode on the Data page. Note the increased signal-to-noise ratio (SNR) in receiver #4 due to close proximity to the MRS voxel position compared to receiver #1, which was furthest away. (**b**) The quality assessment (QA) tool allows signal visualization before and after alignment of individual repetitions with the Align tool. (**c**) Example of four different averaging strategies and their impact on SNR and full width at half maximum (FWHM) of the tCr signal at 3.03 ppm measured in the brain at 7 T. Data averaged without any processing resulted in the lowest spectral SNR and the highest FWHM, and the spectrum was out of phase (black). Phase correction for channel combination improved the SNR and FWHM (green). Additional sensitivity-weighting of individual coil channels further improved SNR (red). The best spectral quality was achieved by also correcting for relative phase and frequency variations between scan repetitions (blue).

INSPECTOR can visualize and selectively analyze data from each coil channel and repetition, or any combinations thereof. This enables inspection of all recorded signals in time or frequency domain for consistency and identification of signals that might be corrupted and therefore should be omitted from the final averaged spectrum. The signals can be visualized individually, as a superposition or as an array (Fig. 3a).

The QA tool and the Align tool work together to support data visualization both before and after the potential corrections of frequency, phase and amplitude. The example shows amplitude (Series mode) as well as phase and frequency (Superposition mode) of the real-valued N-acetylaspartate (NAA) peak measured in the human brain at 7 T before and after frequency and phase alignment with the Align tool. The data can be inspected in series or as a superposition of traces over a user-defined frequency range (Fig. 3b) as well as a spectral array (not shown).

INSPECTOR allows various steps to be involved in the signal processing, from simple averaging of all signals to more refined handling of all signals, which consist of RF receive channel-specific, i.e. relative, phase correction, weighting of receivers and phase and frequency alignment of the resultant spectra (Fig. 3c). As can be seen in the example, the highest SNR and lowest full width at half maximum (FWHM) of the methyl group of total creatine (tCr, 3.03 ppm) signal were measured in a spectrum processed with receiver-specific relative phase correction and sensitivity-weighted averaging, and phase and frequency alignment of the resultant sequence repetitions. Combination of these methods is recommended^9^.

### Processing page

The Processing page contains methods for loading, visualizing, calibrating, filtering, baseline-correcting, and otherwise post-processing spectra.

The data can be transferred from the Data page (resulting from the pre-processed and averaged set of experimental spectra), Synthesis page, or LCM page or directly loaded as INSPECTOR’s native (.mat) data format, the combination of text (.txt) and parameter (.par) files used by the NMRWizard software^19^; data (.raw), parameter (.coord) and basis set (.basis) formats used by LCModel software^20^ and jMRUi’s (.mrui) data format^21^, enabling side-by-side software comparison and interfacing. INSPECTOR uses established methods for signal processing in MRS: Lorentzian and Gaussian line-broadening filters, zero-filling, zero- and first-order phasing, amplitude scaling, frequency shifting, and baseline offsets as described in the literature^22^.

MRS spectra with non-flat baselines can be corrected with the Baseline handling tool. For example, non-flat spectral baselines can originate from solids (e.g., plastics) in the RF coil housing^23^, especially when the data are measured with pulse-acquire methods. The Baseline handling tool allows baseline manipulation using polynomial interpolation over specific frequency ranges. It also allows removing unwanted signals (e.g., water in case of insufficient water suppression) using the Hankel singular value decomposition method^24^.

Standard processing approaches are extended by unique functionalities and algorithms specifically developed for in vivo MRS. For instance, the alignment of two spectra, representing, for example, the condition-specific averages of the JDE spectra ON and OFF, can be achieved by nonlinear least squares optimization considering up to 8 different aspects of spectral processing such as relative frequency shifting, exponential line broadening, polynomial baseline, and others. This advanced spectral alignment can be applied for arbitrary selections of specific frequency ranges over which signals are expected to remain unaffected and can therefore support spectral alignment between different JDE conditions. All these functionalities can be conveniently selected and combined through the GUI.

Analysis of SNR, FWHM and peak integral can be performed over any user-defined target frequency range. Noise for SNR measurement is similarly calculated over a user-defined frequency range.

### Example: *J*-difference editing on GABA at 3 T

**Figure 4:**
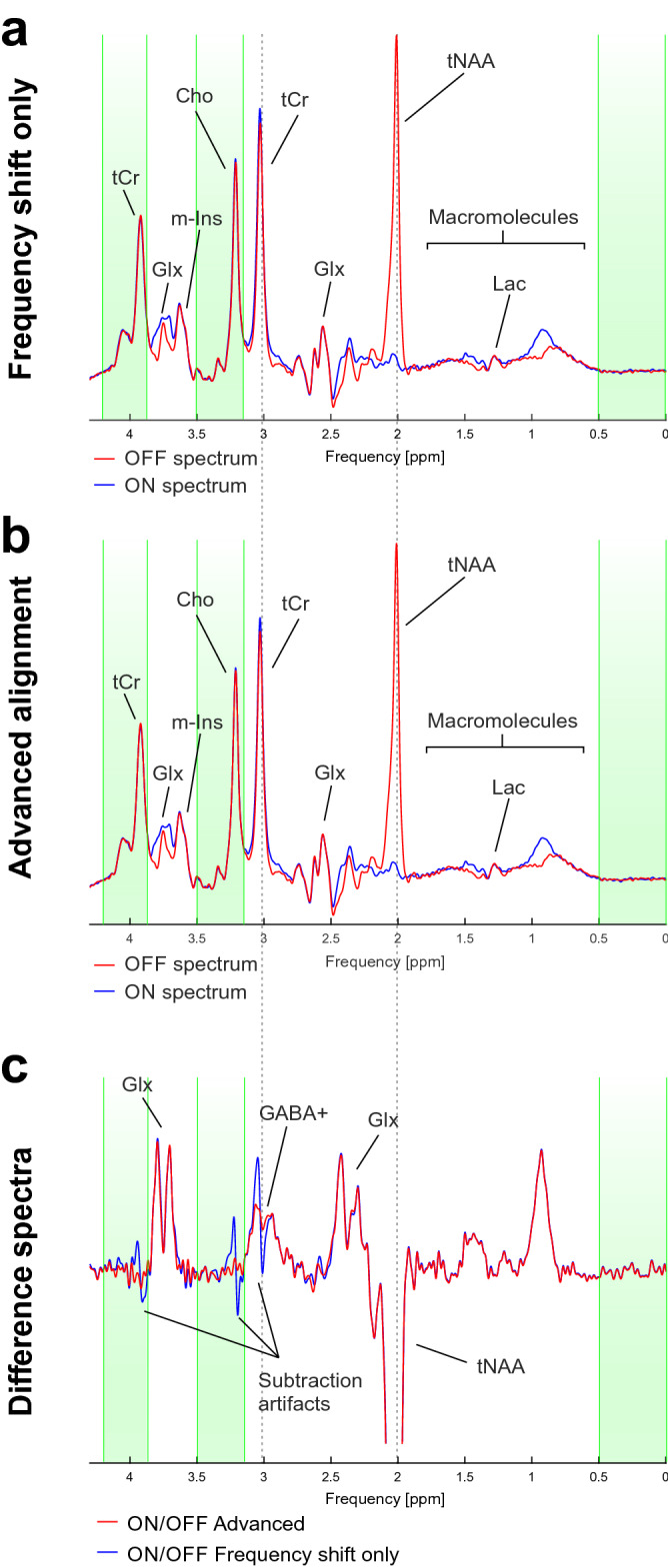
Spectra from *J*-difference editing experiment for GABA acquired from auditory cortex in a healthy adult at 3 T with chosen frequency ranges for alignment shown as green vertical areas. (**a**) Spectra aligned only for frequency shift (blue: ON, red: OFF). (**b**) Spectra with advanced alignment (blue: ON, red: OFF). (**c**) The difference spectrum based on frequency alignment alone (blue) shows severe subtraction artifacts despite a lack of large dissimilarities between the two conditions (**a**), whereas the difference spectrum with advanced alignment (red) is clean and free of subtraction artifacts.

In this example dataset, showing processing of two spectra for measuring glutamate and glutamine (Glx) and gamma-aminobutyric acid (GABA) in a healthy adult (44 years, male) in the auditory cortex, the frequency alignment between averaged JDE ON and OFF spectra concomitantly considering the frequency ranges 0…0.5 ppm, 3.15…3.50 ppm and 3.87…4.20 ppm resulted in visually overlapping and aligned spectral peaks (Fig. 4a,b). Yet the difference between the two conditions exhibited notable subtraction artifacts in spectral areas comprising strong singlet peaks from tCr and choline (Cho) (Fig. 4c, blue). While the subtraction error at 3.20 ppm from Cho is likely irrelevant for quantification, the corresponding tCr-related artifact at 3.03 ppm overlaps with the targeted GABA signal and is thus expected to induce systematic errors in the quantification of GABA. The additional consideration of line broadening, (minute) amplitude scaling, offset and zero-order phase correction, in addition to frequency alignment, not only improves the apparent overlap of spectral ON and OFF conditions (Fig. 4b), but, more importantly, also enables difference signals void of apparent subtraction artifacts (Fig. 4c, red) as a prerequisite for meaningful quantification of GABA from these spectral data.

### Synthesis page

The Synthesis page contains methods for simulating and processing singlet arrays and brain spectra with varying spectral quality and baseline effects.

Basis functions consisting of singlets are useful for LCM analysis of water, lipids, macromolecules or X-nuclei (non-^1^H nuclei) MR spectra. One or more singlets can be simulated according to Larmor frequency (the scanner B_0_ field), bandwidth, calibration of synthesizer frequency in ppm and number of complex points. Every singlet can be individually characterized by frequency in ppm units, amplitude in arbitrary units and linewidth in hertz units, in the MATLAB syntax: [frequency amplitude linewidth]. In addition, complex Gaussian noise and polynomial baselines can be added to the simulated spectrum in the frequency domain in a flexible and well-defined fashion. Signals can be further processed with the same methods as on the Processing page, such as cut, zero filling, Lorentzian and Gaussian line broadening, and corrections to phase, scale, shift, and offset. These functionalities can be used to illustrate and quantify the impact of spectral quality including line shape, baseline and SNR on the accuracy of spectral fitting and linear combination modeling (compare Monte-Carlo functionality on LCM page).

In certain situations, a high-quality brain spectrum can be useful, for example, for SNR simulations, the explorations of the effects of signal processing or for teaching purposes. In this case, a ^1^H MR spectrum can be synthesized on the Synthesis page. This spectrum includes all relevant metabolite signals at (average) concentrations taken from the literature^6^.

### Example: Synthesis page

**Figure 5:**
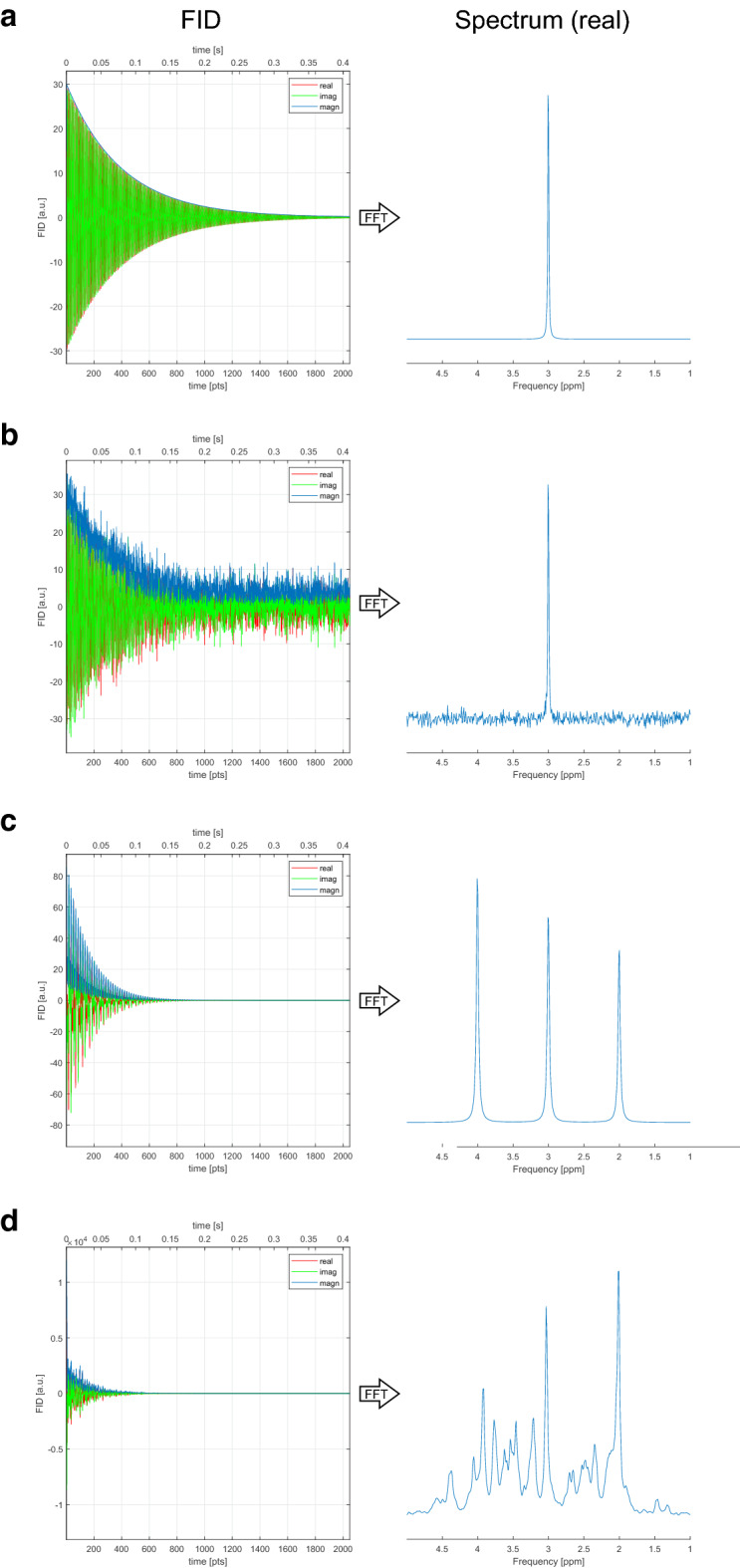
Example generation of singlet and other spectral signals with INSPECTOR. (**a**) Simulated free induction decay (FID) of a singlet [frequency:3 amplitude:30 linewidth:4] with corresponding peak in the frequency domain. The singlets were simulated with Larmor frequency of 298.1 MHz, synthesizer calibrated on 4.65 ppm, bandwidth of 5 kHz and 2048 complex points. (**b**) Adding noise (FID noise = 0.05) to this signal illustrates the concept of signal-to-noise ratio and its impact on MRS data quantification. In this case, the SNR was 62. (**c**) Simulation of multiple singlets ([2 25 10], [3 30 10], [4 35 10]) can help explain different frequencies in the FID and chemical shifts in the frequency domain. (**d**) A synthetic brain spectrum can be generated from readily available metabolite-specific 7 T spectral basis functions incorporated into INSPECTOR to demonstrate the effect of noise on spectral appearance and quantification of spectral signals resembling in vivo conditions.

The Synthesis page allows the generation of singlet and other spectral signals (Fig. 5a), which can be employed as basis functions (or part thereof) for quantification of MRS signals. Adding varying levels of well-defined noise to these signals can be useful for studying the impact of noise for analysis (Fig. 5b). The Synthesis page also enables simulation of singlet arrays (Fig. 5c) and a ^1^H brain spectrum using already simulated basis functions (Fig. 5d), resembling realistic use cases.

### MARSS page

The Magnetic Resonance Spectrum Simulator (MARSS) page contains methods for simulating the signals of arbitrary MRS metabolites, i.e., spin systems, based on the quantum mechanical density-matrix formalism providing a complete theoretical description of any nuclear magnetic resonance experiment^25^.

MARSS has been shown to provide experimentally realistic spectra for the commonly employed MRS sequences STEAM^26^, PRESS^27^, semi-LASER^28^, LASER^29^, and SPECIAL^30^. LCM for quantification employing simulated basis functions relies on the assumption that the calculated spectra resemble physical reality. MARSS has been incorporated into INSPECTOR for the automated simulation of full metabolite basis sets comprising realistic sequence- and vendor-specific spectral shapes for metabolite quantification free of systematic errors based on the selection of scanner vendor, MRS sequence type, and basic acquisition parameters such as B_0_ field strength or T_E_. It has been previously demonstrated that the basis functions expected for even the same sequence by different vendors can differ significantly^10^; as such, the ability to easily generate vendor-specific basis sets, for instance, for Siemens, General Electric and Philips, is of great importance to the MR spectroscopy field.

MARSS is able to simulate even complicated nuclear spin systems more quickly than other available simulation packages^10^ by employing the so-called T-matrix algorithm^31^, which reduces the computational complexity of the task. The current implementation is capable of simulating a full basis set for a three-pulse sequence comprising 23 standard brain metabolites including glucose, GABA and other coupled spin systems with 128^3^ spatial points in 26 min on a personal desktop computer. The theoretical background for MARSS and further performance metrics are described in more detail in the literature^10^.

### Example: simulation of lactate and the superposition of glutamate and glutamine at 3 T

**Figure 6:**
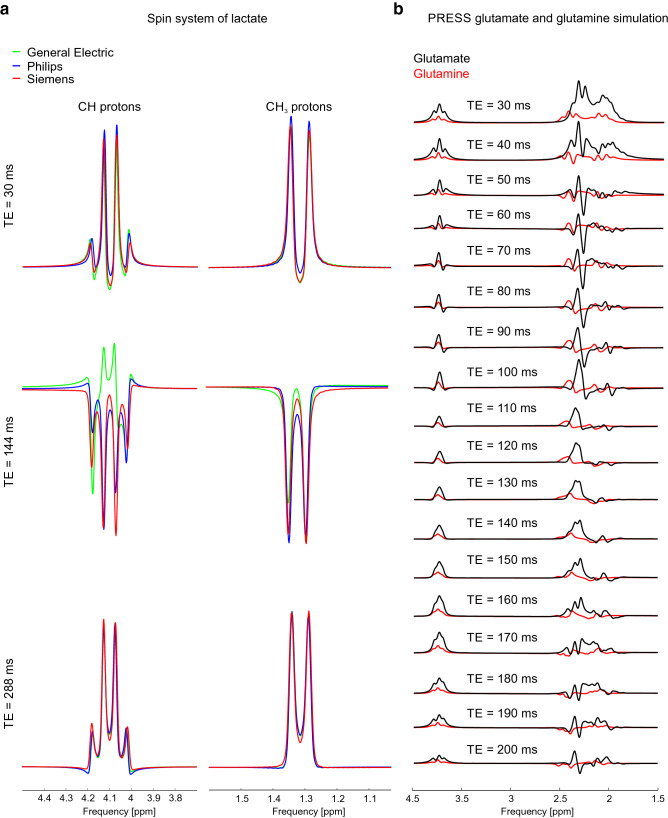
Simulations in MARSS. (**a**) Simulation of lactate molecule for a PRESS sequence by three different vendors and three different echo times for 3 T. Spectra were line broadened with a 1-Hz Gaussian filter. Note that the amplitudes of CH and CH_3_ protons are scaled for display purposes. (**b**) Simulation of glutamate (10.2 mM) and glutamine (2.5 mM) molecules for the realization of the PRESS sequence by Siemens at 3 T over a wide range of echo times. Spectra were line broadened with a 5-Hz Lorentzian filter.

Varying MRS sequence parameters like T_E_ or mixing time (T_M_), and, as mentioned, even vendor-specific implementations of the identical MRS sequence and parameters, have been shown to yield markedly different MRS signal shapes^10^. This can be illustrated using the lactate ^1^H spectrum, which exhibits multiplet resonances from spin-coupled nuclei. Since vendors use different excitation and, more importantly, refocusing RF pulse shapes with different bandwidths, the resultant spectral patterns obtained experimentally will differ depending on these conditions (Fig. 6a). Variations between spectra are small at short T_E_ (30 ms), but they become clearly visible at long T_E_ (144 ms). Such differences can be propagated over a larger basis set of brain metabolites and will inevitably lead to systematic errors in LCM fits and ultimately inaccurate metabolite concentration estimates.

Spectral shapes rely in a complex fashion on MR sequence parameters and details of the spin system such as chemical shift and *J*-coupling. The optimization of spectral shapes for SNR and quantification accuracy as a result of spectral distinctiveness through variation of experimental parameters (e.g., T_E_) is therefore a very time-consuming, cumbersome and costly task when approached experimentally. If the characteristics of the spin system are well known, simulations provide an easy and fast way to observe resolution and potential for separation at various B_0_ field strengths. For example, a well-known problem of effective spectral resolution of glutamate (Glu) and glutamine (Gln) signals at 3 T can be approached by simulating the molecules’ *J*-modulation across various echo times (Fig. 6b).

### LCM page

The linear combination modeling (LCM) page contains methods for loading and processing averaged spectra, creating and managing basis sets, adjusting and running LCM analysis, and exporting results for further data or statistical analysis.

Data for LCM analysis can be retrieved from the Data page, Processing page, or Synthesis page; it can also be directly imported from file in INSPECTOR’s native format (.mat), as text (.txt) and parameter (.par) formats used by NMRWizard software^19^, in the (.raw) data format used by used by LCModel software^20^, and the jMRUI data format (.mrui)^21^.

A Basis Tool is provided to import, visualize, annotate and manage basis functions and create basis sets. Any number of spectral signals can be converted into a basis set, including those generated on the Synthesis page, simulated with MARSS or provided by external software tools such as NMRWizard (.txt, .part), LCModel (.raw) or jMRUI (.mrui).

INSPECTOR’s LCM functionality treats the quantification problem as a nonlinear least squares problem with user-defined tolerance functions, maximum iterations, and maximum function evaluations. It hereby provides access to all parameters and boundary conditions for maximal transparency, i.e. no black box, and flexible user-defined tailoring of the quantification algorithm. LCM can perform quantification of real- or complex-valued spectral shapes including Lorentzian and Gaussian line shaping or combination thereof (Voigt model). The spectral baseline can be handled under full user control including polynomials up to 10^th^ order, b-splines of customizable knot interval and smoothing weight^32^, experimentally measured macromolecule signal(s), broad synthesized signals, or various combinations thereof.

INSPECTOR’s LCM functionality provides Cramér–Rao lower bounds (CRLB)^33^ errors for all optimization parameters including amplitudes (or concentrations), various forms of line broadening, and frequency shifts as well as polynomial and spline baseline shapes, i.e. not only for metabolite concentrations. In addition, CRLB can be calculated for arbitrary metabolite combinations including, but not limited to NAA + N-acetylaspartylglutamate (NAAG), Glu + Gln or choline + phosphocholine + glycerophosphorylcholine (Cho + PCho + GPC). In addition, extended confidence metrics including Monte-Carlo simulations and Hessian error estimates of the least-squares optimization are available for a more complete picture. The full correlation matrix entailing all parameters and calculation of CRLB for any desired metabolite combination (= summation) is supported through flexible adaptation of the prior-knowledge matrix P^33^. In general, INSPECTOR can analyze spectra from any nuclei, B_0_ strength or tissue type, if provided a suitable basis set.

### Example 1: LCM in the brain

**Figure 7:**
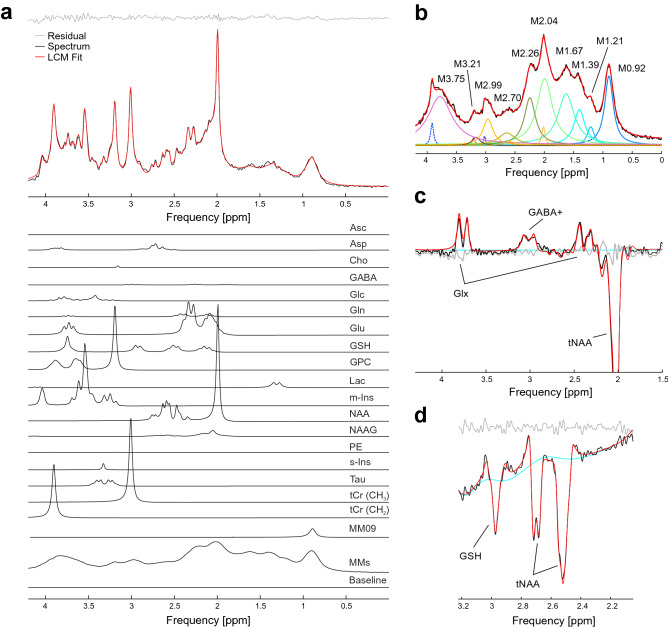
Linear combination modeling (LCM) analysis of in vivo ^1^H MR spectra of the human brain. The LCM fit of all signals is in red. (**a**) Spectrum from medial prefrontal cortex with fit and scaled basis functions. The measured macromolecular signal was included in the basis set. The “MM09” basis function compensated offsets caused by macromolecular signal variation due to differences in T_1_ relaxation times among different macromolecules. Data were measured in a healthy adult at 3 T^3^. (**b**) Analysis of macromolecule spectrum measured in the medial prefrontal cortex at 3 T^43^. The spectrum was obtained from the occipital lobe of a healthy volunteer and shows 10 broad macromolecule resonances (solid colored lines), along with 3 residual singlet resonances assigned to the metabolites NAA (orange), creatine (blue), and choline (purple). (**c**) *J*-difference edited spectrum for gamma-aminobutyric acid (GABA) quantification from the medial prefrontal cortex measured at 3 T (unpublished data). GABA+ denote possible macromolecular contamination due to co-editing effect. (**d**) *J*-difference edited spectrum for glutathione (GSH) from the prefrontal cortex resembling the first successful implementation of MEGA semi-LASER at 7 T^42,44^.

The quantification of brain metabolites can be streamlined with LCM in INSPECTOR employing basis sets generated in MARSS. Short-T_E_ brain spectra (20–30 ms) measured at 3 T contain several overlapping and *J*-coupled signals; therefore, the basis functions of all metabolites and macromolecules need to be simulated with precision or measured using the same sequence as the in vivo data to which the model is being fit. In current practice^8^, the basis functions of metabolites are simulated and the basis function or background signals of macromolecules is measured when applicable. LCM finds appropriate line broadening parameters, frequency shifts, scaling amplitudes, and other applicable transformations for each basis function. The fit with all basis functions, baseline and residual signals can be visualized after the LCM analysis^3^ (Fig. 7a), transferred to other INSPECTOR pages for further analysis or exported as complex FIDs as needed.

Measured macromolecule spectra can not only inform models for quantification of brain metabolites but can also themselves serve as targets for quantification. Analysis of macromolecules can provide insight into metabolism of proteins in brain tissue^34,35^. Since “macromolecules” in MRS parlance comprise what can be large and complex polypeptides with highly complex coupling networks^35^, it is currently not feasible to simulate their spectral shapes from the density matrix formalism like metabolites. It is more common to employ, rather, a basis set consisting of singlets representing groups of known macromolecular signals at specific chemical shifts^36^ (Fig. 7b).

The in vivo detection and measurement of GABA^37^, the major inhibitory neurotransmitter in the mammalian brain, is of particular interest in psychiatry and neuroscience^38,39^. Because signals from GABA overlap with signals of higher-concentration molecules (e.g., tCr at 3.03 ppm), the JDE technique is routinely used for its isolation and detection. However, due to an imperfect profile of editing pulses and similar *J*-coupling constants, GABA is often co-edited with macromolecules (GABA+). Since the editing conditions in the sequence are known, the basis set can be used for analysis of the edited spectrum containing GABA+ , glutamate + glutamine (Glx) and total N-acetylaspartate (tNAA) (Fig. 7c).

Glutathione (GSH) is an endogenous antioxidant implicated in several biological processes, including those associated with multiple sclerosis^40^. Its detection, similarly to GABA, is performed with JDE experiments, which results in an edited spectrum containing GSH and tNAA signals^41^ (Fig. 7d). It has been shown previously that processing and quantification of GSH with INSPECTOR was accurate and reproducible^41,42^.

### Example 2: LCM in preclinical research and X-nuclei MRS

**Figure 8:**
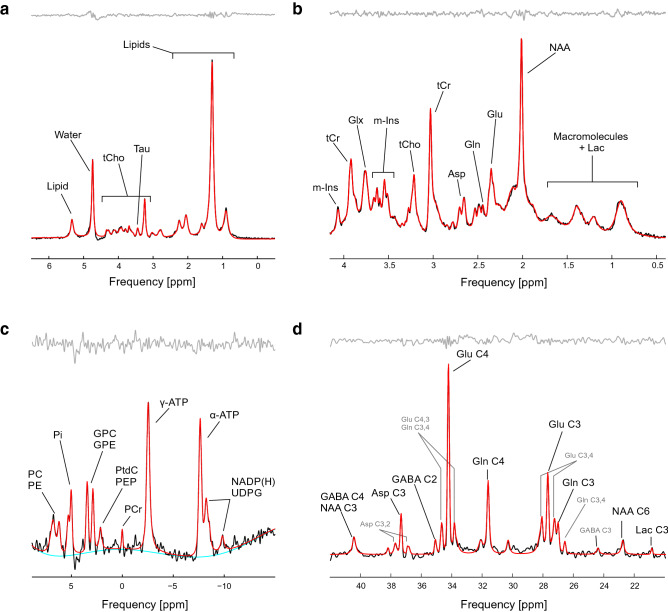
LCM analysis (red) of preclinical and X-nuclei spectra acquired in vivo (black). (**a**) Spectrum from pancreatic tumor in mouse measured at 9.4 T. The basis set consisted of lipid signals, water, total choline-containing compounds (tCho) and taurine (Tau) (Courtesy of Drs. Ken Olive and Yanping Sun, Columbia University, unpublished data). (**b**) Fit of a macaque monkey brain measured at 7 T^36^. Note that the resolution allowed visible separation of Gln and Glu signals at 2.35 and 2.45 ppm. (**c**) ^31^P spectrum measured in the human liver at 7 T^4^ (Courtesy of L. Pfleger et al., Medical University of Vienna). (**d**) ^13^C spectrum from human brain measured at 4 T (Courtesy of Dr. Graeme F. Mason, Yale University, unpublished data). Full list of abbreviations of metabolites is provided in Supplementary Information (Table 1).

Applications of MRS in preclinical oncology research are driven by the detection of chemical compounds associated with cancer biology, for instance choline-containing compounds or total choline (tCho), with a distinctive peak at 3.2 ppm. tCho contains choline, phosphocholine (PC), and glycerophosphocholine (GPC) contributions; however, due to their close chemical shifts, they cannot be resolved from each other using in vivo ^1^H MRS. A spectrum measured from a 0.027 mL volume of interest (VOI) in a murine pancreatic tumor at 9.4 T employed a basis set created in MARSS for LCM quantification of tCho, lipids and taurine (Tau) (Fig. 8a).

The use of short 10 ms T_E_ minimizes signal losses due to T_2_ relaxation effects and increases the contributions of coupled spin systems such as glutamate (Glu) and glutamine (Gln). An analyzed spectrum from a 0.125-mL brain VOI in an anesthetized macaque measured at short T_E_ using a vertical 7 T MR system^36^ showed well resolved Glu and Gln signals at 2.35 ppm and 2.45 ppm, respectively (Fig. 8b).

A prominent disadvantage of ^1^H MRS is the relatively large spectral overlap of metabolites. For example, as mentioned before, the contributions in tCho cannot be well resolved with ^1^H MRS. However, other nuclei, like phosphorus (^31^P), can detect several choline compounds with larger spread in their respective chemical shifts. ^31^P MRS is also often used for investigations into energy metabolism due to relatively easy detection of adenosine triphosphate (ATP)^45^. A ^31^P spectrum measured in the human liver at 7 T was analyzed in LCM with a basis set created on the Synthesis page (Fig. 8c). Note the clear separation of PC and GPC.

Carbon (^13^C) MRS can provide measurements of neuroenergetics and neurotransmitter cycling in the human brain^46^. Thanks to a large spread of chemical shifts, molecules like GABA, Gln and Glu can be detected directly, without strong overlaps (Fig. 8d). Similar to the ^31^P MRS liver example, the basis set in Fig. 8d was created using the Synthesis page.

## Discussion

This paper has provided an overview of the INSPECTOR software and its functionalities, which offer the tools necessary for inspecting, processing and analyzing spectroscopy data in a unitary software solution.

INSPECTOR is intended for users ranging from novices to experts. It is GUI-based and platform-independent, able to read almost all single-voxel MRS data formats. Having all necessary tools in one application, researchers can analyze experimental data right out of the box. Therefore, INSPECTOR can be easily employed by a researcher or laboratory processing and analyzing MRS data for the first time. Experienced users can analyze every trace of the FID via the GUI and discard or fix corrupted signals. This functionality can help to ensure high-quality experimental data and to troubleshoot if needed, e.g. in case of coil hardware failure or subject motion. Each functionality can be fine-tuned according to specific experimental needs. High-level data processing and quality management were incorporated into the pipeline to ensure data consistency and reproducibility.

INSPECTOR is written in MATLAB, which gives users several advantages. All spectral data can be exported in a MATLAB data format, thereby rendering them easily accessible for additional processing if needed. Since all figures are displayed in MATLAB, graphics can be exported in various graphical formats (e.g., .png, .tif, .eps), which enables easy handling and publication. The software includes ample options for documentation and visualization, including automated figure export in the above graphical formats or saving of LCM results in MS Excel file tables.

Protocol files are an important feature of INSPECTOR. MRS data need to be treated differently according to the tissue, nuclei, and acquisition methods from which they were derived. Each experiment requires unique settings of numerous parameters, which can be difficult to memorize and recover, a method prone to human errors. Protocol files save all this information in a complete image of the current software setup that can be shared across experiments, users or laboratories. Therefore, data measured at different sites can be processed and analyzed in a coherent and even fully identical fashion, ensuring consistency and scientific rigor. Protocol files can serve as backups for experimental settings as well.

Simulations can help to answer MRS-related research questions, such as: estimation of SNR, optimal T_E_ for measurement of specific *J*-coupled metabolites, proper sequence parameters for JDE experiments, or prototyping novel sequences prior to implementation on the scanner. This approach can deliver results relatively quickly and without frequent and costly proof-of-concept scans.

The combination of in-depth and reproducible data visualization, processing, and simulation make INSPECTOR an ideal teaching tool^47^. Complete visualization of experimental data, simulations, and signal analysis enable review and discussion in a classroom setting, both in-person and remote. Protocol files with stored parameters can help instructors to share a MRS problem to solve, guide students in their approaches, and furthermore allow students to save and share their solutions. With the focus on teaching, it is hoped that students from any background can learn and use MRS methods via INSPECTOR with ease and interest, possibly contributing to future expansion of the in vivo MRS community.

INSPECTOR is a software tool available to the academic MRS community free of charge. Software support is provided by members of MR SCIENCE Laboratory at Columbia University (http://juchem.bme.columbia.edu). The laboratory organizes annual workshops with focus on both in vivo MRS methodology and application. Moreover, INSPECTOR has its own user community accessible by the LinkedIn social network. The MRS laboratories of several universities already use INSPECTOR and, to date, the software has been downloaded more than 200 times.

Future extensions of INSPECTOR include batch processing and spectroscopic imaging functions. Both are already implemented and regularly applied in our laboratory. These functionalities, once validated across vendor-provided data formats, will be made available with a future release. 2D NMR methods are not supported in the current version of INSPECTOR.

The INSPECTOR software package for MRS spectroscopy data inspection, processing, simulation and analysis was presented. Ease of use, compatibility with other data formats and data quality control from loading the data to export of analytical results make INSPECTOR a software of choice for wide use of the MRS methods in preclinical and clinical research.

### Software availability

INSPECTOR has been available for download under a non-commercial license free of charge since 2017 at: http://innovation.columbia.edu/technologies/cu17130_inspector.

For academic use, this work should be cited. For commercial use or source code request, please contact us directly.

## Supplementary Information

Supplementary Information
