# Supplementary material for: INSPECTOR: free software for magnetic resonance spectroscopy data inspection, processing, simulation and analysis
Source: Sci Rep. 2021 Jan 22;11:2094. doi: 10.1038/s41598-021-81193-9 (PMC7822873; doi:10.1038/s41598-021-81193-9)
Supplement: Supplementary file 1 — Supplementary Information [file 41598_2021_81193_MOESM1_ESM.docx]

**Supplementary Information to**

**INSPECTOR: Free Software for Magnetic Resonance Spectroscopy Data Inspection, Processing, Simulation and Analysis**

Martin Gajdošík^1^, Karl Landheer^1^, Kelley M. Swanberg^1^, and Christoph Juchem^1,2^

**﻿***^1^Department of Biomedical Engineering, Columbia University Fu Foundation School of Engineering and Applied Science, New York, NY*

*^2^Department of Radiology, Columbia University College of Physicians and Surgeons, New York, NY*

**S1: Abbreviations of Metabolites**

Metabolically interesting nuclei for *in vivo* MRS include proton (^1^H), carbon-13 (^13^C) and phosphorus (^31^P). Each nucleus can provide a wealth of metabolic information which can be studied non-invasively with MRS. ^1^H MRS can detect neurotransmitters (glutamate, GABA) and other compounds^1^. ^13^C MRS offers the possibility to study metabolic pathways (tricarboxylic acid cycle or Krebs cycle) or detect glycogen for instance^2^. ^31^P MRS provides information about energetically important metabolites, intracellular pH, and reaction fluxes^3^. An extensive list of metabolites measurable with *in vivo* MRS can be found in the literature^4^.

Table 1 lists several cerebral metabolites and compounds found in pancreatic and liver tissues that are abundant in the example spectra presented in the paper.

**Table 1**: Names of metabolites and groups of metabolites with their corresponding abbreviation and nucleus/nuclei by which these metabolites are typically measured.

| **Metabolite or Group of Metabolites** | **Abbreviation** | **Nucleus** |
| --- | --- | --- |
| Adenosine triphosphate | ATP | ^31^P |
| Ascorbate | Asc | ^1^H |
| Aspartate | Asp | ^1^H |
| Choline | Cho | ^1^H |
| Choline + Glycerophosphorylcholine | tCho | ^1^H |
| Creatine | Cr | ^1^H |
| Creatine + Phosphocreatine | tCr | ^1^H |
| γ-Aminobutyric acid | GABA | ^1^H, ^13^C |
| γ-Aminobutyric acid + macromolecules | GABA+ | ^1^H |
| Glucose | Glc | ^1^H, ^13^C |
| Glutamate | Glu | ^1^H, ^13^C |
| Glutamine | Gln | ^1^H, ^13^C |
| Glutamate + Glutamine | Glx | ^1^H |
| Glutathione | GSH | ^1^H |
| Glycerophosphoethanolamine | GPE | ^31^P |
| Glycerophosphorylcholine | GPC | ^31^P |
| Glycine | Gly | ^1^H |
| Lactate | Lac | ^1^H |
| Macromolecules | MM | ^1^H |
| Myo-inositol | m-Ins | ^1^H |
| N-acetylaspartate | NAA | ^1^H |
| N-acetylaspartylglutamate | NAAG | ^1^H |
| N-acetylaspartate + N-acetylaspartylglutamate | tNAA | ^1^H |
| Nicotinamide adenine dinucleotide-phosphate | NADP(H) | ^31^P |
| Phosphate (inorganic) | Pi | ^31^P |
| Phosphatidylcholine | PtdC | ^31^P |
| Phosphocreatine | PCr | ^31^P, ^1^H |
| Phosphocholine | PC | ^31^P |
| Phosphoenolpyruvate | PEP | ^31^P |
| Phosphorylethanolamine | PE | ^31^P |
| Scyllo-inositol | s-Ins | ^1^H |
| Taurine | Tau | ^1^H |
| Uridine diphosphate-glucose | UDPG | ^31^P |

**S2: Overview of INSPECTOR’s Pages and Functionalities**

Each page in INSPECTOR consist of functions tailored for specific processing step. Overview of the functions with their short description is in Table 2.

**Table 2:** Quick summary of INSPECTOR functionalities.

| **Page** | **Function** | **Description** |
| --- | --- | --- |
| Data | Data selection | Data can be loaded as a single file or as selection of files. |
|  | Data handling and visualization | Visualization of all acquired signals in real, imaginary, magnitude or phase mode. |
|  | Coil channel combination | Selection and weighting of coil channels. |
|  | Phase correction | Klose method or based on the 1^st^ point of the reference signal. |
|  | QA tool | Visualization of spectral repetitions. |
|  | Align tool | Frequency, phase and/or, amplitude alignment of repetitions. |
| Processing | Spectral processing | Apodization, zero filling, Lorentzian and Gaussian line broadening, corrections of zero and first order phase, scale, shift, offset. |
|  | Frequency calibration | Assign known frequency to a peak. |
|  | Processing of 2 spectra | Advanced alignment methods, superposition, summation and difference of 2 spectra. |
|  | Peak analysis | Calculation of SNR, |
|  | Baseline handling | Baseline corrections and peak(s) removal. |
| Synthesis | Simulate singlets | Simulate singlet or multiple singlets. |
|  | Simulate noise  Simulate baseline | FID noise power per square root of bandwidth.  Simulate zero through 5^th^ order polynomial spectral baseline. |
|  | Synthesize brain | Simulation of a brain spectrum from provided basis set. |
|  | Spectral processing | Apodization, zero filling, Lorentzian and Gaussian line broadening, corrections of phase, scale, shift, offset. |
|  | Peak analysis | Calculation of SNR, FWHM and peak integral. |
| MARSS | Sequence selection | STEAM, PRESS or custom sequence. |
|  | Origin / Vendor | General Electric, Siemens or Philips. |
|  | Parameter definition | Either T_E_ (and T_M_ for STEAM) for stock sequences, input directly via GUI or .mat file for custom sequences |
|  | Spin system library | List of all spin systems implemented into INSPECTOR. |
|  | Basis selection | List of spin systems which will be simulated by MARSS. |
|  | Simulation details | B_0_ field, bandwidth, complex points, Lorentzian line broadening, Larmor frequency, dimensions, T_E_ and T_M_ (in case of STEAM) |
|  | Spectral processing | Apodization, zero filling, Lorentzian and Gaussian line broadening, corrections of phase, scale, shift, offset. |
| LCM | Manage | Tool for creation and management of basis sets. |
|  | Frequency window(s) | User-defined frequency range(s) for signal quantification. |
|  | Spectral and basis set processing | Apodization, zero filling, Lorentzian and Gaussian line broadening, corrections of phase, scale, shift, offset. |
|  | Frequency window(s) | User defined frequency range(s) for signal quantification. |
|  | LCM analysis | Linear combination model analysis in real or complex mode. |
|  | Fit details | Starting values, lower and upper bounds used for LCM. |
|  | MC simulations | Monte-Carlo error analysis. |
|  | Correlation matrix | Display and save correlation matrix of all model parameters. |
|  | LCM traces | Visualization of individual, grouped or all fitted LCM traces. |
|  | Save .xls | Save all fitting results to a MS Office Excel spreadsheet file. |

**S3: Acquisition Details**

MRS spectra presented in the paper were measured with various localization sequences and parameters (Table 3). The technical overview of single-voxel sequences and chemical shift imaging (CSI) sequences can be found in the literature^5,6^.

**Table 3:** Sequences and their parameters used for measurement of spectra showed in the examples.

| **Figure** | **Sequence** | **T_E_ [ms]** | **T_R_ [s]** | **T_M_ [ms]** | **N_A_** | **VOI [mm^3^]** | **B_0_ [T]** | **Reference** |
| --- | --- | --- | --- | --- | --- | --- | --- | --- |
| 3 a,b,c | STEAM | 10 | 3 | 50 | 170 | 20x20x20 | 7 | Juchem et al.^7^ |
| 4 a.b.c | MEGA-sLASER | 72 | 2 | NA | 256 | 25x25x40 | 3 | Unpublished |
| 7 a | sLASER | 20 | 2 | NA | 32 | 25x25x25 | 3 | Landheer et al.^8^ |
| 7 b | sLASER | 20 | 2 | NA | 128 | 25x25x25 | 3 | Landheer et al.^9^ |
| 7 c | MEGA-sLASER | 72 | 2 | NA | 256 | 25x25x40 | 3 | Unpublished |
| 7 d | MEGA-sLASER | 72 | 3 | NA | 128 | 30x30x30 | 7 | Swanberg et al.^10^ |
| 8 a | PRESS | 16.3 | 1.7 | NA | 256 | 3x3x3 | 9.4 | Unpublished |
| 8 b | STEAM | 10 | 4 | 10 | 256 | 5x5x5 | 7 | Pfeuffer et al.^11^ |
| 8 c | 3D-CSI | 1.1 | 1.5 | NA | 1 | 200x200x200* | 7 | Pfleger et al.^12^ |
| 8 d | ISIS | <5** | 2.5 | NA | 640 | 50x40x45 | 4 | Unpublished |

*The spectrum was created by averaging of spectra from selected voxels from a 16x16x16 matrix.

** This ISIS sequence with polarization transfer was optimized to detect the CH_2_ groups of glutamate and glutamine.

**REFERENCES**

1. Govindaraju, V., Young, K. & Maudsley, A. A. Proton NMR chemical shifts and coupling constants for brain metabolites. *NMR Biomed* **13**, 129–153 (2000).

2. Rothman, D. L., de Feyter, H. M., de Graaf, R. A., Mason, G. F. & Behar, K. L. 13C MRS studies of neuroenergetics and neurotransmitter cycling in humans. *NMR Biomed.* **24**, 943–957 (2011).

3. Valkovič, L., Chmelík, M. & Krššák, M. In-vivo 31P-MRS of skeletal muscle and liver: A way for non-invasive assessment of their metabolism. *Anal. Biochem.* (2017) doi:10.1016/j.ab.2017.01.018.

4. de Graaf, R. A. *In Vivo NMR Spectroscopy: Principles and Techniques 3rd Edition*. (John Wiley & Sons, Ltd, 2019). doi:10.1002/9781119382461.

5. Landheer, K., Schulte, R. F., Treacy, M. S., Swanberg, K. M. & Juchem, C. Theoretical description of modern 1 H in Vivo magnetic resonance spectroscopic pulse sequences. *J. Magn. Reson. Imaging* jmri.26846 (2019) doi:10.1002/jmri.26846.

6. Maudsley, A. A. *et al.* Advanced magnetic resonance spectroscopic neuroimaging: Experts’ consensus recommendations. *NMR Biomed.* 1–22 (2020) doi:10.1002/nbm.4309.

7. Juchem, C. & de Graaf, R. A. B 0 magnetic field homogeneity and shimming for in vivo magnetic resonance spectroscopy. *Anal. Biochem.* **529**, 17–29 (2017).

8. Landheer, K., Gajdošík, M. & Juchem, C. A semi-LASER, single-voxel spectroscopic sequence with a minimal echo time of 20.1 ms in the human brain at 3 T. *NMR Biomed.* e4324 (2020) doi:10.1002/nbm.4324.

9. Landheer, K., Gajdošík, M., Treacy, M. & Juchem, C. Concentration and effective T2 relaxation times of macromolecules at 3T. *Magn. Reson. Med.* mrm.28282 (2020) doi:10.1002/mrm.28282.

10. Swanberg, K. *et al.* Towards in vivo neurochemical profiling of multiple sclerosis with MR spectroscopy at 7 Tesla: Cross-sectional assessment of frontal-cortex glutathione, GABA, and glutamate in individuals with relapsing-remitting and progressive multiple sclerosis. in *ISMRM* #2970 (2017).

11. Pfeuffer, J., Juchem, C., Merkle, H., Nauerth, A. & Logothetis, N. K. High-field localized 1H NMR spectroscopy in the anesthetized and in the awake monkey. *Magn. Reson. Imaging* **22**, 1361–1372 (2004).

12. Pfleger, L. *et al.* Absolute Quantification of Phosphor-Containing Metabolites in the Liver Using 31 P MRSI and Hepatic Lipid Volume Correction at 7T Suggests No Dependence on Body Mass Index or Age. *J. Magn. Reson. Imaging* **c**, (2018).
